# An interdisciplinary approach for laparoscopic removal of a large retroperitoneal pelvic schwannoma attached to vital vessels: A case report

**DOI:** 10.1097/MD.0000000000018149

**Published:** 2019-12-20

**Authors:** Antonio Macciò, Paraskevas Kotsonis, Laura Aste, Maria Andreea Voicu, Clelia Madeddu, Carlo Conti, Stefano Camparini

**Affiliations:** a Department of Gynecologic Oncology Surgery; b Department of Neurosurgery, Azienda Ospedaliera Brotzu; c Department of Medical Sciences and Public Health University of Cagliari; d Department of Vascular Surgery, Azienda Ospedaliera Brotzu, Cagliari, Italy.

**Keywords:** laparoscopy, large abdominal vessels, minimally invasive surgery, retroperitoneal schwannoma

## Abstract

**Rationale:**

Retroperitoneal schwannomas are very rare and may grow very close to major abdominal vessels. Since the surgical approach to the retroperitoneal space may be complex due to surrounding vital organs, including major vessels, laparoscopic surgery is challenging and has only been recently adopted. Here, we report a case of laparoscopic resection of a large retroperitoneal schwannoma attached to large vital vessels.

**Patient concerns:**

A 62-year-old woman presented with a chief complaint of pain in the lower right limb with consequent claudication, which had lasted for approximately 1 year.

**Diagnoses:**

Magnetic resonance imaging revealed a solid oval mass measuring 45 × 32 × 39 mm, located medially to the right iliopsoas muscle at the level of the intersomatic space between the 5th lumbar vertebra and the 1st sacral vertebra. This mass was inhomogeneously hypointense in T2 due to the presence of cystic areas, with intense and inhomogeneous contrast enhancement, compatible with the diagnosis of a schwannoma. The mass compressed the inferior caval vein near its bifurcation and the right common iliac vein, anteriorly dislocating the ipsilateral iliac arterial axis.

**Interventions:**

A multidisciplinary team skilled in vascular and pelvic laparoscopy was involved. The patient underwent laparoscopic surgery via an anterior transperitoneal approach with right adnexectomy and radical excision of the tumor. The surgery lasted 120 minutes without intraoperative complications. Blood loss was less than 100 mL. The histologic diagnosis was a benign Schwannoma; grade I according to World Health Organization classification.

**Outcomes:**

The postoperative course was uneventful. At the 10-month follow-up, the patient had no recurrences and was asymptomatic.

**Lessons:**

Laparoscopic removal of large retroperitoneal schwannomas, even if attached to major vital vessels, is feasible and safe when performed by experienced surgeons.

## Abstract 2

Supplemental Digital Content is available in the text

## 1 Introduction

Schwannomas are typically benign tumors belonging to the class of soft-tissue neoplasms, which derive from the Schwann cells of the peripheral nerve envelope.^[1]^ Usually, they are encapsulated well-circumscribed masses that displace, rather than invade, local structures. Schwannomas rarely (incidence of 0.7%–2.7%) occur in the retroperitoneal space,^[2]^ where they typically grow close to major vital vessels. There are usually asymptomatic and radiological features are often not well-defined. Therefore, regardless of the use of advanced imaging technologies, preoperative diagnosis of retroperitoneal schwannomas is very difficult, and biopsy is not recommended owing to the elevated risk of complications.^[3]^ Surgical radical removal is the preferred treatment and is associated with low incidence of relapses.^[4]^ Since surgical approach to the retroperitoneal space may be complex due to surrounding organs, such as the pancreas and major vessels, open surgery has been widely used.^[5]^ However, more recently, laparoscopic surgery has been adopted for retroperitoneal schwannomas. Here, we report a case of successful radical removal of a large retroperitoneal schwannoma, which was adherent to the inferior caval vein bifurcation, via anterior laparoscopic surgery.

## 2 Case report

**Figure 1:**
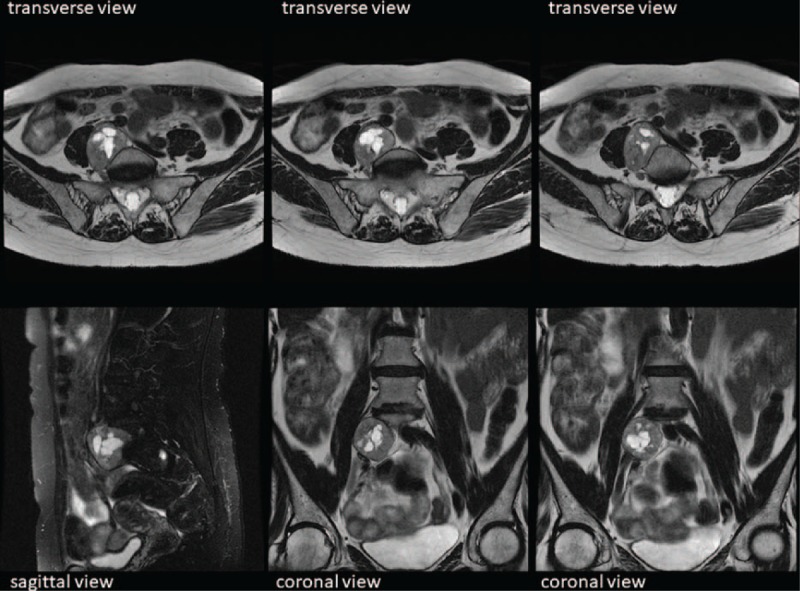
Magnetic resonance imaging. Transverse, sagittal, and coronal scan showing a well-demarcated retroperitoneal mass anterior to the soma of the 5^th^ lumbar vertebra, attached to the inferior vena cava and the right common iliac vein, and anteriorly dislocating the ipsilateral iliac arterial axis.

A 62-year-old woman (body mass index: 24) was referred to the Neurosurgery Department (in our hospital) with a 1-year history of pain in the right lower limb with consequent claudication. On physical examination, her abdomen was nontender with no palpable mass. An abdominal ultrasound revealed a suspected mass at the right iliopsoas muscle level. Computed tomography showed a solid tumor anterior to the soma of the 5th lumbar vertebra, compressing the right iliac vessels. Magnetic resonance imaging (Fig. 1) revealed a solid oval mass, measuring 45 × 32 × 39 mm, localized medially to the right iliopsoas muscle at the level of the intersomatic space between the 5th lumbar vertebra and the 1st sacral vertebra. This mass was inhomogeneously hypointense in T2 due to the presence of cystic areas, with intense and inhomogeneous contrast enhancement, compatible with the diagnosis of a schwannoma. This tumor affected the anterolateral right profile of the L5 soma, had a mass effect on the inferior caval vein near its bifurcation and on the right common iliac vein, and anteriorly dislocated the ipsilateral iliac arterial axis. The lesion did not invade spinal nerves roots. Laboratory test findings were normal, including serum levels of C-reactive protein, fibrinogen, and tumor markers (carcinoembryonic antigen, Cancer antigen [CA]-125, CA 19.9).

Due to the tumor location, attachment to vital vessels, and proximal location to pelvic organs (right adnexa and ureter), the neurosurgeons, aiming to perform a minimally invasive surgery, decided to set up a multidisciplinary team experienced in vascular and pelvic laparoscopy. The team comprised a neurosurgeon, vascular surgeon, and an oncological gynecology team. We decided to perform laparoscopic surgery using an anterior transperitoneal approach with right adnexectomy and radical excision of the tumor. The patient provided written informed consent both for the surgery and for the publication of the case report and related images. The retrospective study design did not require approval but only notification to the local ethics committee.

## 3 Surgical technique

Surgery was performed with the patient under general anesthesia in the supine position. Considering the risk of complication, especially that of vessel damage, all laparoscopic instruments (grasper, vascular clips and clamps, bipolar instruments) for managing vascular injury were available in the operating room. Moreover, we also prepared all instruments that are useful for conversion to open surgery (abdominal laparotomy) in the event of severe injury and uncontrollable bleeding. Further, blood packs for transfusion were available before surgery was commenced.

We used 4 trocars based on the body constitution. The first 10 to 12 mm optical trocar was placed in the periumbilical area and a 10 mm 0°-degree laparoscope was introduced through it. Next, a 5-mm trocar was placed in the left hypochondrium, lateral to the rectus abdominis muscle. Another 5-mm trocar was placed in the suprapubic position, and a fourth 10 to 12 mm trocar was placed at the right hypochondrium lateral to the rectus abdominis muscle. A pneumoperitoneum of 10 to 15 mm Hg was obtained and maintained throughout the surgery.

First, we performed right adnexectomy according to standard laparoscopic procedure to enable better visualization of the surgical plane and identification of the right ureter and right iliac vessels (Supplementary Video S1, online only [Supplementary Video S1]. Video showing laparoscopic radical removal of a large retroperitoneal schwannoma, which is attached to major abdominal vital vessels [video velocity 4x]). Next, we proceeded with the isolation of the ureter and identification of the right hypogastric nerve. Subsequently, we incised the peritoneum up to the aortic bifurcation following the course of the right iliac artery. Thereafter, to improve management in the event of vascular injury and bleeding, we isolated the right external iliac vein and the right common iliac artery with vessel loops, removing the adherent lymphatic tissue. The vena cava and its bifurcation, the aortic bifurcation, and the internal iliac artery (anterior and posterior branches) were also accurately visualized. The tumor was detected in the retroperitoneal space below the inferior caval vein bifurcation and the right iliac common vein, lateral to the right internal iliac vein. The internal iliac vein was wrapped tightly around the tumor. We carefully detached the mass from the vessels by using endoscopic 5-mm blunt cherry dissectors (Ethicon Endopath, Hamburg, Germany), with gauze dissection. Next, the capsulated mass was radically excised without complications. Hemostasis was controlled, and after careful washing, a TABOTAMP (Ethicon) was placed and stuffed at the implantation bed of the excised neoplasm. We used LigaSure Small Jaw Open Sealer/Divider (Covidien, Boulder, CO) for all surgical procedures. We extracted the excised mass by the 12-mm trocar with a surgical bag (Endocatch, Ethicon). A closed suction drain was placed in the surgical bed for 24 hours. The surgery lasted 120 minutes without intraoperative complications. Blood loss was less than 100 mL. The removed mass measured 50 × 40 × 30 mm. Histologic and immunohistochemistry diagnosis was a benign Schwannoma, grade I according to World Health Organization classification with strong and diffuse expression of the neurogenic marker, S 100 protein, and rare mitoses. The postoperative course was uneventful, and the patient was discharged on the 2nd postoperative day. At the 12-month follow-up, the patient had no recurrences and was asymptomatic.

## 4 Discussion

Schwannomas are usually asymptomatic tumors with gradual growth, whose diagnosis is often incidental. The retroperitoneal location is associated with its large size (>3 cm), which results in close relationship and strong adherence to large abdominal vessels.^[6]^ Our paper describes a case of a large retroperitoneal Schwannoma that was successfully excised via a laparoscopic anterior transperitoneal approach. To the best of our knowledge, since only a few cases of large retroperitoneal schwannomas have been reported in the literature, guidelines on the optimal surgical treatment are lacking. The surgical approach must be discussed on a case-by-case basis with respect to the surgeon's experience, the lesion's characteristics, and the involvement of abdominal structures and vessels.^[7]^ With recent progress in minimally invasive surgery, being the surgeons more skilled with the development of novel laparoscopic devices, the indication for laparoscopy has widened and laparoscopic surgeries for retroperitoneal schwannomas have been reported.^[8–22]^ Specifically, a search of the English literature revealed less than 10 cases of successful laparoscopic resection of schwannomas that were adherent to large vessels.^[14–22]^ Among these, the largest mass was reported by Petrucciani et al^[14]^ and measured 9.5 × 4.4 × 2.5 cm; it was very adherent to the inferior vena cava. This mass was laparoscopically removed with the patient placed in the left lateral decubitus position. Descazeaud et al^[15]^ described the largest schwannoma excised via an anterior laparoscopic approach. The schwannoma measured 8 × 5 cm and compressed the inferior vena cava. Other cases of retroperitoneal schwannoma compressing large vital vessels, which were successfully removed via an anterior laparoscopic approach, ranged from 2.9 × 2.7 × 2.5 cm to 5.5 × 3.5 cm in diameter.^[16–20]^ Gorgun et al^[21]^ reported another case of a large retroperitoneal schwannoma, measuring 4.5 × 5.2 cm, compressing the inferior vena cava, which was laparoscopically removed with the patient in the left lateral decubitus position. Ohsawa et al^[22]^ described laparoscopic excision of a retroperitoneal schwannoma (4.0 × 1.7 cm) adjacent to iliac vessels (internal iliac artery), which was performed with the patient in the dorso-sacral position.

A retrospective study demonstrated that laparoscopy in comparison to open surgery was associated with a shorter postoperative hospital stay.^[23]^ Considering the possibility of benign schwannoma relapse, the main goal of surgery is to achieve complete excision, preserving neuronal and other intra-abdominal structures.^[13,24]^ Further, it should be highlighted that in our case, the tumor was adherent to adjacent vital large vessels, that is, the inferior caval vein bifurcation, common iliac vein, and right internal iliac vein. Moreover, the proximity of the tumor to the ureter was carefully considered. For this reason, laparoscopic excision of such a large retroperitoneal schwannoma was very difficult and required skill and competence, both for laparoscopic and vascular surgery. In our case, the tumor was radically excised with careful identification, preparation, and dissection of the vascular planes. In this regard, visual magnification of the surgical field provided by the anterior laparoscopic approach guarantees a safe and precise surgery,^[19]^ allowing an excellent view of the relationship between the tumor and surrounding vital structures that are more difficult to visualize with the conventional posterior approach.^[25]^ Additionally, employing the latest appropriate technical tools was very useful in performing the delicate steps of the mini-invasive approach successfully. Thus, laparoscopic resection based on oncological surgical principles was possible.

In conclusion, it should be noted that laparoscopic approach is feasible and can allow radical removal of retroperitoneal schwannomas with minimal invasiveness. This is an important factor in deciding the optimal surgical approach since this neoplasm commonly affects young individuals and this population may benefit from laparoscopy, which has the benefits of rapid recovery and favorable cosmetic results.

## Acknowledgment

The authors thank Concetta De Simone for her technical assistance.

## Author contributions

**Conceptualization:** Antonio Macciò, Clelia Madeddu, Carlo Conti, Stefano Camparini.

**Data curation:** Antonio Macciò, Paraskevas Kotsonis, Laura Aste, Maria Andreea Voicu, Carlo Conti, Stefano Camparini.

**Formal analysis:** Antonio Macciò, Laura Aste, Clelia Madeddu, Stefano Camparini.

**Funding acquisition:** Antonio Macciò.

**Investigation:** Antonio Macciò, Paraskevas Kotsonis, Maria Andreea Voicu, Clelia Madeddu, Carlo Conti, Stefano Camparini.

**Methodology:** Antonio Macciò, Paraskevas Kotsonis, Laura Aste, Maria Andreea Voicu, Clelia Madeddu, Carlo Conti, Stefano Camparini.

**Resources:** Antonio Macciò, Laura Aste, Clelia Madeddu, Carlo Conti.

**Supervision:** Antonio Macciò, Clelia Madeddu.

**Validation:** Antonio Macciò, Clelia Madeddu.

**Writing – original draft:** Antonio Macciò, Laura Aste, Maria Andreea Voicu, Clelia Madeddu, Carlo Conti, Stefano Camparini.

**Writing – review and editing:** Antonio Macciò, Paraskevas Kotsonis, Laura Aste, Maria Andreea Voicu, Clelia Madeddu, Carlo Conti, Stefano Camparini.

## Supplementary Material

Supplemental Digital Content
